# The First Complete Chloroplast Genome Sequence of the *Cyrtomium hemionitis* Fern

**DOI:** 10.3390/cimb47090771

**Published:** 2025-09-18

**Authors:** Junxi Zhao, Panpan Shi, Xiaoxuan Wang, Shuosheng Zhang, Haixian Zhan

**Affiliations:** Institute of Pharmaceutical & Food engineering, Shanxi University of Chinese Medicine, Jingzhong 030619, China; zhaojunxitj@126.com (J.Z.); 11111120231208@163.com (P.S.); 15602019431@163.com (X.W.); zhangshuosheng@aliyun.com (S.Z.)

**Keywords:** *C. hemionitis*, chloroplast genome, phylogenetic relationship, comparative genomics

## Abstract

*Cyrtomium hemionitis* is a *Cyrtomium* fern with potential medicinal value; however, the lack of chloroplast genome data for this species limits its utilization and exploitation. In this study, the Illumina NovoSeq 6000 platform and SPAdes v3.14.1 were used to sequence and assemble the chloroplast genome of *C. hemionitis*. The chloroplast genome was 151,295 bp in length and exhibited a typical circular, double-stranded, quadripartite plastome architecture, with a GC content of 42.43%. Additionally, it included 30 high-frequency codons, 26 of which ended with A or U. In total, we annotated 130 coding genes, which included 88 protein-coding genes, 8 rRNA genes, and 34 tRNA genes. The IR (inverted repeat) boundaries of the genus *Cyrtomium* differed from those of common plants, with differences discovered in the JLB (large single-copy, inverted repeat b) and JLA (large single-copy, inverted repeat a) boundaries in this genus. Additionally, the phylogeny of this genus showed that *C. hemionitis* was more closely related to *C. falcatum*, whereas *Dryopteris crassirhizoma* was closely related to the genus *Cyrtomium*. These findings have significant implications for future research and can serve as a reference for the molecular evolution, systematic development, and utilization of *C. hemionitis*.

## 1. Introduction

*C. hemionitis*, a perennial herbaceous plant of the Dryopteridaceae family, is widely distributed in temperate regions, being mainly located in the southern Guizhou and Yunnan provinces of China [1]. *Cyrtomium* plants have high medicinal value in Chinese Traditional Medicine (CTM), given their antiviral, anthelmintic, and hemostatic properties. *Cyrtomium fortunei*, in particular, is efficacious in clearing heat and dampness, cooling blood to stop bleeding, and killing insects [2]. Moreover, past research has demonstrated the effects of Grandiflora *Cyrtomium*, specifically that the ethanol extract of this plant ameliorates the immunosuppressive effect of cyclophosphamide in mice [3]. Other research indicated that *Dryopteris fragrans* extract has an inhibitory effect on skin fungi [4]. Additionally, *D. crassirhizoma* polysaccharides were found to possess antiviral properties, indicating their potential for applications in food and pharmaceutical products [5]. However, in recent years, habitat degradation and anthropogenic destruction have reduced the range of the *Dryopteridaceae* family, particularly affecting species with very small populations, including *C. hemionis*. Therefore, it is imperative to implement conservation efforts for the germplasm resources of *C. hemionitis*.

Chloroplasts serve as crucial sites for plant photosynthesis and play an important role in plant growth and development. Studying chloroplast genome sequences is essential for uncovering genetic diversity, interspecific relationships, and the evolutionary adaptability of plants. A chloroplast genome is characterized by its simple structure and small molecular weight, and it follows a matrilineal inheritance pattern [6]. Chloroplast genomes have been used to identify ferns with phylogenetic and basal origins such as *Dryopteris crassirhizoma* [7], *Neolepisorus fortunei* [8], and *Cyrtomium fortunei* [2]. Consequently, chloroplast genomes have great potential for application in the field of molecular marker development and in the identification of closely related species.

In this study, we employed bioinformatics methods to analyze the chloroplast genome of *C. hemionitis*, thereby elucidating its gene structure and clarifying its evolutionary relationships within the genus *Cyrtomium*. These findings may be valuable for the future development of molecular markers and phylogenetic studies of *C. hemionitis*, as well as the conservation and utilization of the germplasm resource.

## 2. Materials and Methods

### 2.1. Plant Materials, DNA Extraction, and Sequencing

**Figure 1:**
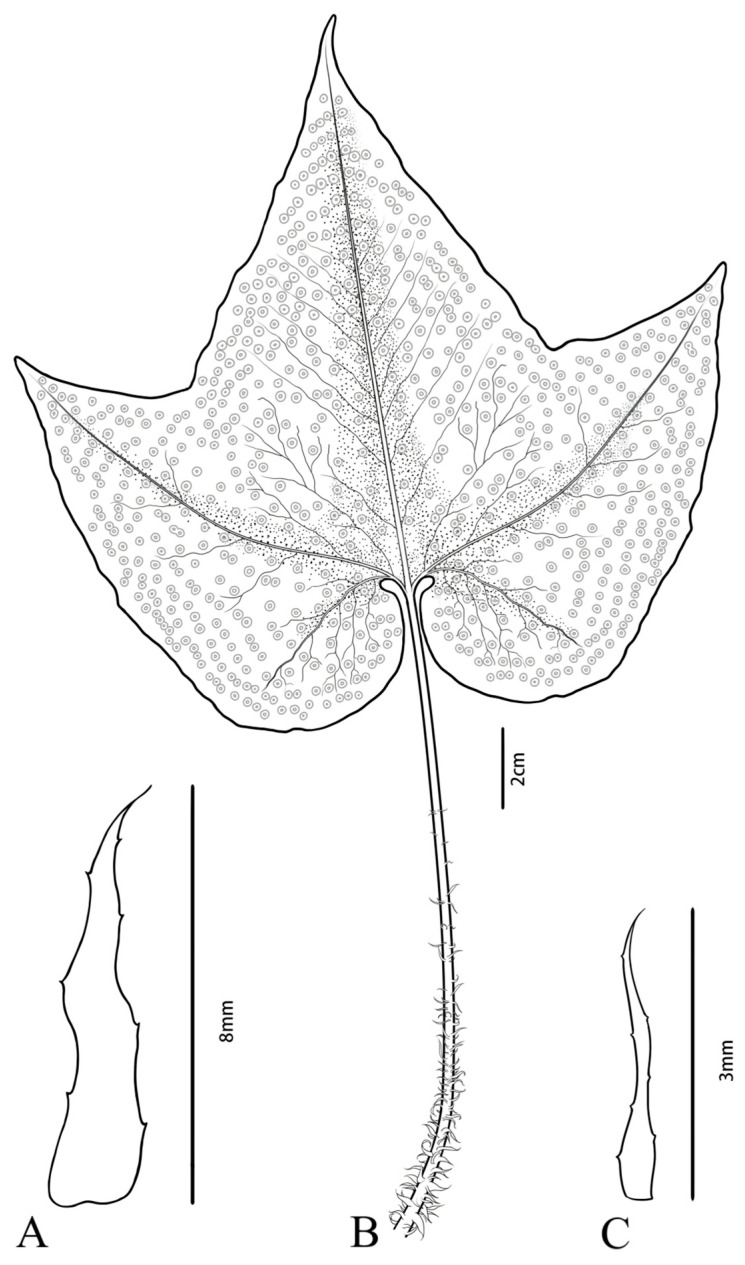
*C. hemionitis*. Note: (**A**) Petiole basal scales; (**B**) leaves and petioles without lateral pinnae; (**C**) middle petiole scale (drawn by H. Y. Chen and X. R. Chen).

A specimen of *C. hemionitis* was collected by Panpan Shi on 4 May 2025, in Wenshan Zhuang and Miao Autonomous Prefecture, Yunnan Province, China (104°50′28.14″ E, 23°10′0.9″ N). To ensure accuracy, the material was identified as *C. hemionitis* by Chunfa Chen, an assistant researcher at Lushan Botanical Garden, Jiangxi Province, and the Chinese Academy of Sciences. The specimen was preserved in the Specimen Room of the Shanxi University of Chinese Medicine (SXTCM), under the accession number 20250504. The morphology of this plant is shown in Figure 1. Total DNA of the healthy leaves was extracted via the SDS method [9], and DNA quality was measured via 1% agarose gel electrophoresis and ultraviolet spectrophotometry (concentration 723.9 ng/µL, A260/A280 = 1.89). We constructed libraries with an average length of 350 bp using the Nextera XT DNA Libraries Preparation Kit (Illumina, San Diego, CA, USA), and the libraries were then sequenced on the Illumina NovaSeq 6000 platform.

### 2.2. Genome Assembly and Annotation

Fastp software (version 0.19.7) was used for quality control processing of the raw sequence reads [10], with the raw data totaling 6.05 G and the clean data totaling 5.99 G after quality control processing. The GC content of the clean data was 44.94%, the average sequencing depth was 878.57 X, and the Q30 value was 96.81%, indicating that the quality of the chloroplast genome sequencing and assembly results was very high. The high-quality reads were then assembled into the chloroplast genome using de novo assembler SPAdes v.3.14.1 software (Set k-mer 21, 45, 65, 85105) [11], and chloroplast genome annotation was performed using PGA software [12], with manual correction performed on the annotated results. After annotating the sequences, we generated a file and submitted it to the GenBank database (Accession Number: PV990400), using the edited GenBank annotation file to submit OGDRAW [13] and draw the annotation map. The raw sequencing data was uploaded to the SRA database, BioSample ID PRJNA1314546.

### 2.3. Relative Synonymous Codon Usage Analysis

CodonW v1.3 software was used to estimate the relative synonymous codon usage of the codons in the chloroplast genome of *C. hemionitis*. The RSCU (Relative Synonymous Codon Usage) value was evaluated as follows: if the RSCU value was equal to 1, the codon was used without preference, and if the RSCU value was greater than 1, a high-frequency codon was used for the amino acid; otherwise, a low-frequency use codon was used instead [14,15]. RSCU values were not disturbed by amino acid composition and were considered a response to codon usage preference, with RSCU > 1 considered a high-frequency codon [16].

### 2.4. Neutrality Plot, ENC-Plot, and PR2-Bias Plot Analysis

A Neutrality Plot analysis of the codon genes was performed with GC12 (the average of GC1 and GC2) content as the y-axis and GC3 content as the x-axis, based on the genomes obtained. The closer the data points clustered to the diagonal line, the stronger the correlation and the greater the evidence that mutational bias was the dominant factor in codon usage. A random distribution of data points suggested that codon usage preferences were significantly influenced by factors other than mutation, such as natural selection [17]. ENC-Plot analysis was used for scatter plots, with ENC values placed on the vertical axis and GC3 values placed on the horizontal axis. The effects of mutation and selection on codon preference were analyzed by plotting a standard curve, whose formula was ENC = 2 + GC3 + 29/[GC3^2^ + (1 − GC3)^2^]. Codon usage was primarily influenced by mutation when it was close to the neutral curve expected, whereas significant deviations indicated a stronger influence of natural selection [18]. The X-axis and Y-axis represented G3/(G3 + C3) and A3/(A3 + T3), respectively, in the scatter plot of the deviation analysis. Additionally, the center point (A=T, G=C) was used to analyze the magnitude and direction of the basic deviation [19].

### 2.5. Comparative Analysis of Chloroplast Genomes

The online tool IRscope [20] was used to compare the linkage sites between *C. fortunei*, *C. falcatum*, *C. devexiscapulae*, and *C. hemionitis*, with regions including large single-copy (LSC), small single-copy (SSC), and inverted repeat a/b (IR a/b) contractions and expansions.

### 2.6. The Genomic SSR Markers Detection

MISA software was used to identify the microsatellite loci (http://pgrc.ipk-gatersleben.de/misa/misa.html, accessed on 5 September 2025), with the following search parameters set for identification: nucleotide motifs of 2, 3, 4, 5, and 6 nucleotides required at least 6, 5, 4, 4, and 4 repeat sequences, respectively [21].

### 2.7. Phylogenetic Analysis

A phylogenetic tree was constructed using MEGA11 software (maximum likelihood method, bootstrap = 1000) for 10 materials, including 3 *Cyrtomium* species downloaded from NCBI, 5 *Dryopteris* plants, *Lepisorus clathratus* as an outgroup, and the sequenced *C. hemionitis*.

## 3. Results

### 3.1. Characteristics of C. hemionitis Chloroplast Genomes

**Figure 2:**
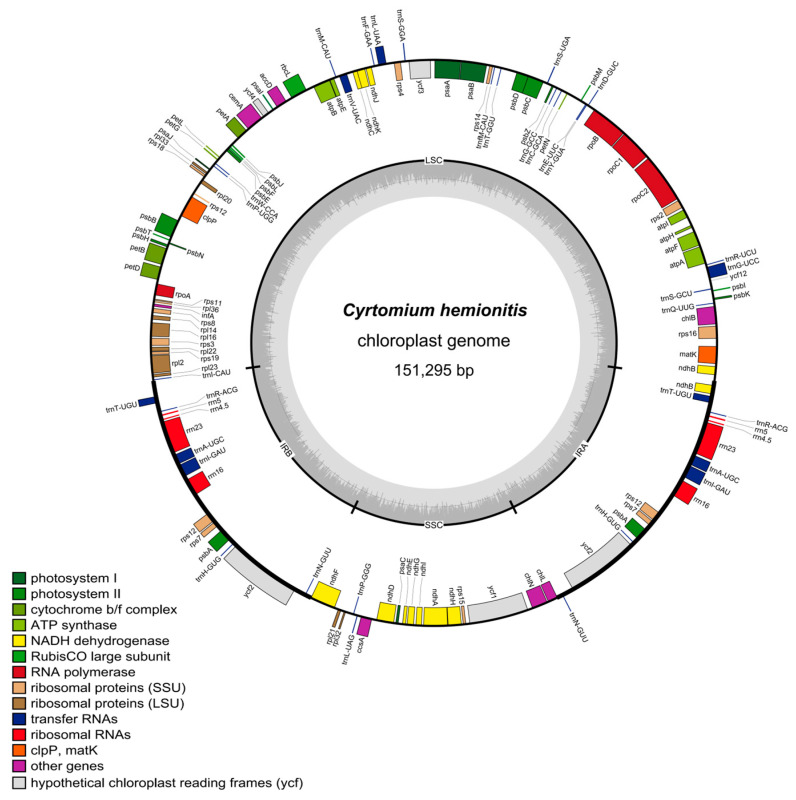
Gene map of the *C. hemionitis* complete chloroplast genomes.

The chloroplast genome of *C*. *hemionitis* was a typical circular, double-stranded molecule with a genome length of 151,295 bp. Similarly to most plants, its chloroplasts were divided into four regions, namely, the SSC region, the IRa region, the LSC region, and the IRb region, with lengths of 21,624 bp, 23,754 bp, 82,163 bp, and 23,754 bp, respectively (Figure 2). The chloroplasts of *C. hemionitis* encoded 130 genes, of which 88 were protein-coding genes, 8 were rRNA genes, and 34 were tRNAs. The *atpF*, *ndhA*, *ndhB*, *petB*, *petD*, *rpl16*, *rpl2*, *rpoC1*, *rps16*, *trnA-UGC*, *trnG-UCC*, *trnI-GAU*, *trnL-UAA*, *trnT-UGU*, and *trnV-UAC* genes each contained one intron, the *clpP* and *ycf3* genes contained two introns, and trans-splicing occurred in the *rps12* gene (Table 1).

### 3.2. Codon Preference Analysis

**Figure 3:**
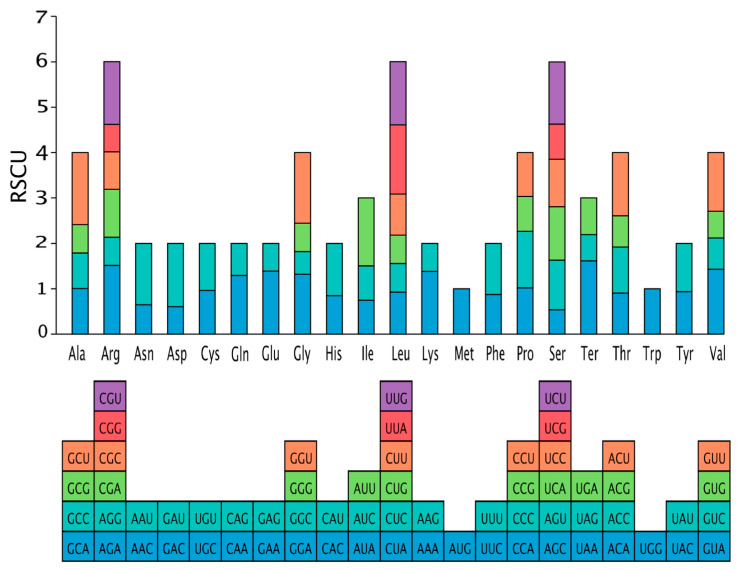
Relative synonymous codon use in the chloroplast genome of *C. hemionitis*.

The codon composition showed that the average content of GC_all_ in the chloroplast genome of *C. hemionitis* was 43.54%, and the GC content of each part was noticeably different, with the average content of GC1 (GC content at codon position 1), GC2 (GC content at codon position 2) and GC3 (GC content at codon position 3) being 49.39%, 42.41%, and 38.80%, respectively, and the lowest content seen in GC3. Thirty codons in the chloroplast genome had an RSCU value greater than 1 (Figure 3), and twenty-six of these ended in A/U, indicating a tendency for A/U-ending codons to feature in *C. hemionitis*. Twenty-eight codons had RSCU values less than 1, showing a weak preference for their use, while only the methionine (AUG) and tryptophan (UGG) codons had a neutral RSCU value of 1.

### 3.3. Codon Usage Bias Analysis

**Figure 4:**
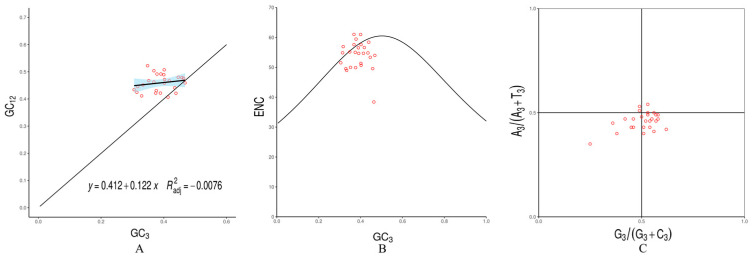
Analysis of codon preference-influencing factors in *C. hemionitis*. (**A**) Neutrality Plot analysis; (**B**) ENC-Plot analysis; (**C**) PR2-Bias Plot analysis. The scattered points in the figure represent genes.

The Neutrality Plot of the *C. hemionitis* chloroplast genome indicated that most genes were located above the diagonal line, with a regression coefficient of 0.122 (Figure 4A). This indicated that the codons were affected by mutations at a rate of 12.2%. ENC-GC3 correlation analysis revealed that the actual ENC values of the vast majority of genes were lower than the expected ENC values (Figure 4B), suggesting that codon bias in the chloroplast genome of *C. hemionitis* was influenced by natural selection. Figure 4C shows that the distribution of genes was uneven across the four quadrants centered at 0.5, with more genes distributed in the lower regions than in the upper regions, and a higher concentration in the lower-right quadrant. The results above indicate that the *C. hemionitis* chloroplast genome exhibited a higher frequency of T>A and G>C base usage, with the third codon position showing a significantly higher frequency of T/G than A/C. This further demonstrated that its codons were significantly influenced by natural selection.

### 3.4. Expansion and Contraction of IRs

**Figure 5:**
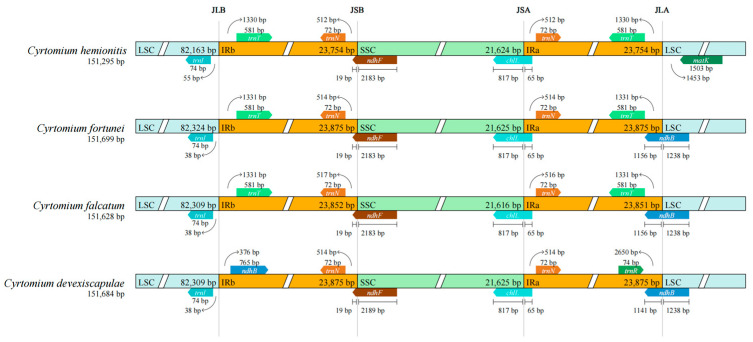
Comparison of the junctions of the LSC, SSC, IRa, and IRb zones in four *Cyrtomium* species.

An analysis conducted on the chloroplast genome SC/IR boundary between *C. hemionitis* and its congeners (Figure 5) revealed that the junctions of all four *Cyrtomium* species were more conservative, especially JSB and JSA. In JSB and JSA of the four species, *ndhF* and *chlL* were located in the middle, respectively, but the degree of expansion was slightly different. The distances between the *ndhF* boundary and the JSB in *C. devexiscapulae* were 2189 bp and 19 bp, respectively, whereas for the other three *Cyrtomium* species, the distances were 2183 bp and 19 bp, respectively. The distances between the *chlL* boundaries and JSA for all four *Cyrtomium* species were 817 bp and 65 bp. The JLB of *C. hemionitis*, *C. fortune*, and *C. falcatum* were all located in the IGS between genes *trnl* and *trnT*, whereas the JLB of *C. devexiscapulae* was located in the IGS between genes *trnl* and *ndhB*. The JLA of *C. hemionitis* was located in the IGS between the *matK* and *trnT* genes, whereas the JLA of the remaining three species was located in the *ndhB* gene, with slightly different degrees of expansion.

### 3.5. Genomic SSR Marker Development

A total of 50 SSR sites were detected in the *C. hemionitis* genome (Table 2). Of these, single nucleotides were the most numerous, with 45, accounting for 90.00% of the total. Four dinucleotides and one tetranucleotide were identified; no other types of nucleotides were detected.

### 3.6. Phylogenetic Relationship Analysis

**Figure 6:**
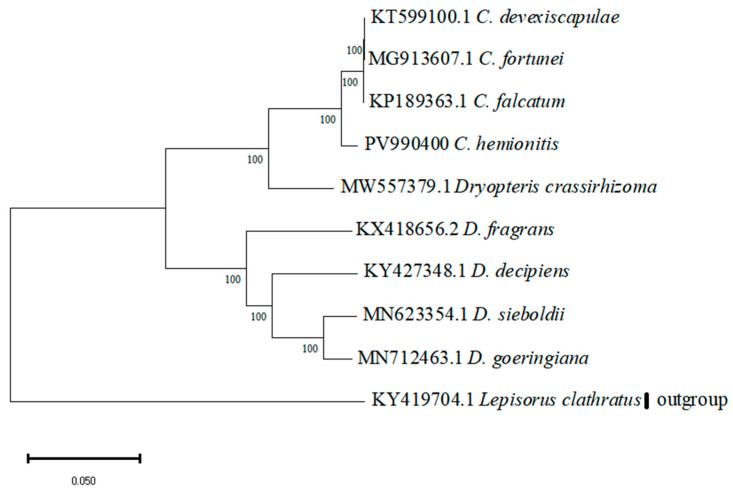
Phylogenetic tree of the chloroplast genome.

In the phylogenetic tree, the Bootstrap Value for all nodes was 100% (Figure 6). The Dryopteridaceae were divided into two sub-branches, one containing four species of *Dryopteris* and the other containing *Dryopteris crassirhizoma*, *C. hemionitis*, *C. falcatum*, *C. fortunei*, and *C. devexiscapulae*. By contrast, the outgroup *Lepisorus clathratus* formed a separate clade.

## 4. Discussion

### Structural Variations in the Chloroplast Genomes of Cyrtomium Species

The chloroplast genome is a typical tetrameric sequence [22] that is relatively conserved in structure, with both (high) similarity and differences between genes mainly originating from intergenic regions. This study was the first to complete the sequencing and analysis of the chloroplast genome of *C. hemionitis*, finding that the chloroplast genome was 151,295 bp in length, with a double-stranded circular tetrad, a GC content of 42.43%, and a chloroplast genome length similar to that of *C. falcatum*, *C. fortunei*, and *C. devexiscapulae*. Moreover, the genome size and structural composition of the *Cyrtomium* genus were highly conserved.

Chloroplast genome codon usage preference is related to gene selection and genetic variation, among others, and it is also closely related to inter-species kinship and habitat status [23]. Research has shown that 96.55% of codons preferentially used by 27 species of Leguminosae end in U/A [24], and, in the chloroplast genome of *C. hemionitis*, 86.66% of the 30 codons used in its preference ended in A/U; apart from these, 28 codons were used with a degree of usage of less than 1, and only AUG and UGG had no preference. This is consistent with the codon usage preferences seen in many ferns [25]. Moreover, Neutrality Plot, ENC-Plot, and PR2-Bias Plot analyses all indicated that codon usage preferences in *C. hemionitis* were primarily influenced by natural selection.

The contraction and expansion of the IR region significantly affects the size of the genome [26], as seen in the IR boundaries of the genus *Cyrtomium*, which were rather unusual, possibly related to its status as a sporophyte. The JSB and JSA boundaries of four species of the genus *Cyrtomium* were located in the *ndhF* and *chlL* genes, respectively. By contrast, these two boundaries are located in the *ycf1* gene in other plants, e.g., *Sapindaceae* [27] and *Canna* [10]. The distribution of the LSC/IRb (JLB) boundaries in species of the genus *Cyrtomium* was more specific, mostly located in the overlapping *trnl* and *trnT* genes, whereas these boundaries were generally located in the *rsp19* gene in other plants, e.g., *Impatiens* [28] and *Arisaema* [29]. The LSC/IRa (JLA) junction in the species of this genus was mostly located in the *ndhB* gene, whereas in other species, this boundary was located in the *trnH* gene [30,31]. Additionally, the phylogenetic tree indicated that *D. crassirhizoma* was more closely related to the genus *Cyrtomium*. Furthermore, *C. hemionitis* was more closely related to *C. falcatum*.

## 5. Conclusions

In this study, the size and structure of the chloroplast genome of *C. hemionitis* were analyzed, with the results demonstrating that this genus exhibited a preference for A/U-terminated codons in its chloroplast genome, consistent with the codon usage preferences observed in most plants. Among the four boundaries of the genus *Cyrtomium*, the most significant variation was identified in the JLA boundary, whereas the other three boundaries exhibited minimal changes. Moreover, phylogenetic analyses revealed that the medicinal plant *D. crassirhizoma* was closely related to *C. hemionitis*, suggesting that *C. hemionitis* may possess potential medicinal value. These findings contribute to the chloroplast genome database and can serve as a valuable reference for molecular biology research on fern species.

## Figures and Tables

**Table 1:** Genetic constitution of the chloroplast genome of *C. hemionitis*.

Category	Gene Group	Gene Name
Photosynthesis	Subunits of photosystem I	*psaA*, *psaB*, *psaC*, *psaI*, *psaJ*
	Subunits of photosystem II	*psbA*(2), *psbB*, *psbC*, *psbD*, *psbE*, *psbF*, *psbH*, *psbI*, *psbJ*, *psbK*, *psbL*, *psbM*, *psbN*, *psbT*, *psbZ*
	Subunits of NADH dehydrogenase	ndhA*, ndhB*, *ndhC*, *ndhD*, *ndhE*, *ndhF*, *ndhG*, *ndhH*, *ndhI*, *ndhJ*, *ndhK*
	Subunits of cytochrome b/f complex	*petA*, petB*, petD*, *petG*, *petL*, *petN*
	Subunits of ATP synthase	*atpA*, *atpB*, *atpE*, atpF*, *atpH*, *atpI*
	Large subunit of rubisco	*rbcL*
	Subunits photochlorophyllide reductase	*chlB*, *chlL*, *chlN*
Self-replication	Proteins of large ribosomal subunit	*rpl14*, rpl16*, rpl2*, *rpl20*, *rpl21*, *rpl22*, *rpl23*, *rpl32*, *rpl33*, *rpl36*
	Proteins of small ribosomal subunit	*rps11*, rps12**(2), *rps14*, *rps15*, rps16*, *rps18*, *rps19*, *rps2*, *rps3*, *rps4*, *rps7*(2), *rps8*
	Subunits of RNA polymerase	*rpoA*, *rpoB*, rpoC1*, *rpoC2*
	Ribosomal RNAs	*rrn16*(2), *rrn23*(2), *rrn4.5*(2), *rrn5*(2)
	Transfer RNAs	trnA-UGC*(2), *trnC-GCA*, *trnD-GUC*, *trnE-UUC*, *trnF-GAA*, *trnG-GCC*, trnG-UCC*, *trnH-GUG*(2), *trnI-CAU*, trnI-GAU*(2), trnL-UAA*, *trnL-UAG*, *trnM-CAU*, *trnN-GUU*(2), *trnP-GGG*, *trnP-UGG*, *trnQ-UUG*, *trnR-ACG*(2), *trnR-UCU*, *trnS-GCU*, *trnS-GGA*, *trnS-UGA*, *trnT-GGU*, trnT-UGU*(2), trnV-UAC*, *trnW-CCA*, *trnY-GUA*, *trnfM-CAU*
Other genes	Maturase	*matK*
	Protease	clpP**
	Envelope membrane protein	*cemA*
	Acetyl-CoA carboxylase	*accD*
	c-type cytochrome synthesis gene	*ccsA*
	Translation initiation factor	*infA*
Genes of unknown function	Conserved hypothetical chloroplast ORF	*ycf1*, *ycf12*, *ycf2*(2), ycf3**, *ycf4*

One asterisk = one intron; two asterisks = two introns.

**Table 2:** Simple sequence repeats of the chloroplast genome in *C. hemionitis*.

Repeats	Total	Proportion/%
A	8	16
C	16	32
G	7	14
T	14	28
AT	3	6
TA	1	2
TCTA	1	2

## Data Availability

The data that support the findings of this study are openly available in GenBank, accession number PV990400.
